# The quality of commercial SARS-CoV-2 nucleic acid tests in Ecuador: lessons from COVID-19 pandemic for advancing social equity through microbiology

**DOI:** 10.3389/fcimb.2023.1179786

**Published:** 2023-06-07

**Authors:** Diana Morales-Jadan, Bernardo Castro-Rodriguez, Carolina Viteri-Dávila, Solon Alberto Orlando, Alfredo Bruno, Franklin Perez, Miguel Angel Garcia-Bereguiain

**Affiliations:** ^1^ One Health Research Group, Universidad de Las Americas, Quito, Ecuador; ^2^ Universidad Espiritu Santo, Guayaquil, Ecuador; ^3^ Universidad Agraria del Ecuador, Guayaquil, Ecuador; ^4^ One Labt, Santa Elena, Ecuador

**Keywords:** SARS-CoV-2, emergency use authorization, Ecuador, RT-q PCR, RT- LAMP, clinical performance

## Introduction

The “coronavirus disease 2019” (COVID-19) pandemic, caused by Severe Acute Respiratory Syndrome Coronavirus 2 (SARS-CoV-2), challenged the public health systems worldwide since the initial outbreak in China in December 2019 (Gorbalenya et al., 2020; Zhou et al., 2020).

The gold standard for the detection of SARS-CoV-2 is the reverse transcription–quantitative polymerase chain reaction (RT-qPCR), although other nucleic acid amplification tests (NAATs) like the reverse transcription–loop-mediated isothermal amplification (RT-LAMP) are also available. There are several available RT-qPCR assays developed by public institutions such as the Centers for Diseases Control and Prevention (CDC, Atlanta, USA) or the Charité Hospital (Berlin, Germany), endorsed by the World Health Organization for SARS-CoV-2 diagnosis (Corman et al., 2020; Lu et al., 2020; PAHO, 2020). However, the COVID-19 pandemic created a huge NAATs demand from laboratories worldwide not ever seen before. Under this scenario, hundreds of NAATs commercial kits for SARS-CoV-2 diagnosis became available during the last 3 years, some of them with Emergency Use Authorization (EUA) by the US Food and Drug Administration (FDA) or other international regulatory agencies, while others have scarce information related to their clinical performance and the lack of EUA at the country of manufacture (https://www.fda.gov/medical-devices/emergency-situations-medicaldevices/emergency-use-authorizations; FIND, 2020). Due to the COVID-19 pandemic, the EUA of SARS-CoV-2 NAATs kits adopted flexible regulatory protocols. Fortunately, the performance of these kits has been evaluated by research groups worldwide with the subsequent publication of their results in scientific journals (Udugama et al., 2020; Hernandez et al., 2021; Yu et al., 2021).

## The weakness of low- and middle-income countries to guarantee the quality of commercial SARS-CoV-2 NAATs: the case of Ecuador

The majority of NAATss for the detection of SARS-CoV-2 are developed by companies from high-income countries. Therefore, middle- and low-income countries have had to import commercial NAATs kits for SARS-CoV-2 diagnosis. For instance, this is the case of countries in South America like Ecuador, where multiple commercial NAATs kits for SARS-CoV-2 produced in the USA, Canada, Europe, China, or South Korea are available. While some of those assays have obtained EUA by reputed governmental agencies in the countries of manufacture, others, for undisclosed reasons, have been denied with a local EUA. Whether with a local EUA granted or not, SARS-CoV-2 NAATs kits have been commercialized in middle- and low-income countries with the understanding that the latter (i.e., EUA not granted) have been unethically distributed. At least in Ecuador, the urgent need for containing the COVID-19 pandemic made public health authorities to exempt SARS-CoV-2 NAATs kits commercialized in the country for presenting an EUA from the country of manufacture. No local experimental evaluation of the clinical performance was required before using the assays for the detection of SARS-CoV-2 in potentially infected patients. This could have generated a country-wide underestimation of the COVID-19 pandemic burden, leading to an increased morbidity and mortality. We emphasize the importance of validating tests with patient samples in the field in the country where those SARS-CoV-2 NAATs were intended to be used. Moreover, we highlight the importance to evaluate the performance of the SARS-CoV-2 NAATs not only prior to their commercialization but also after commercial distribution to follow up that quality was guaranteed.

## The role of academia to support a good-quality SARS-CoV-2 diagnosis in Ecuador

To assure the quality of the clinical performance of SARS-CoV-2 NAATss during the COVID-19 pandemic crisis in Ecuador, research laboratories have been actively conducting evaluation studies of SARS-CoV-2 NAATs kits available in the country. As it is summarized in **Table 1**, a total of 11 SARS-CoV-2 NAATs kits were evaluated in Ecuador (Freire-Paspuel et al., 2022; Freire-Paspuel and Garcia-Bereguiain, 2020; Freire-Paspuel et al., 2020a; Freire-Paspuel and Garcia-Bereguiain, 2021a; Freire-Paspuel and Garcia-Bereguiain, 2021b; Freire-Paspuel and Garcia-Bereguiain, 2021c; Freire-Paspuel et al., 2021; Freire-Paspuel et al., 2021).

**Table 1:** Clinical performance and analytical sensitivity of 11 commercial SARS-CoV-2 NAAT kits available in Ecuador. (EUA is referred to clinical authorization use within country of manufacture and/or by FDA).

Detection kit (company, country)	Sensitivity (manufacturer)	Specificity (manufacturer)	LoD (manufacturer)	Sensitivity (observed)	Specificity (observed)	LoD (viral copies/ml of sample) (copies/µl of RNA extraction)	EUA	Ref.
**nCoV-QS** **(MiCo BioMed, South Korea)**	NA	NA	1.8 copies/µl of RNA extraction	70.6%–100%	92.9%–100%	10,000 50	**Ecuador**	(Freire-Paspuel et al., 2020a; Salinas et al., 2022)
**AccuPower SARS-CoV-2 real time RT-PCR kit (Bioneer, South Korea)**	NA	NA	NA	78.9%–100%*	100%	40,000 200	**Ecuador**	(Freire-Paspuel and Garcia-Bereguiain, 2020; Fellner et al., 2021)*
**AccuPower SARS-CoV-2 Multiplex RT-PCR kit (Bioneer, South Korea)**	100%	100%	2 copies/µl of RNA extraction	73.5%	100%	18,000 90	**Ecuador**	(FIND, 2020; Freire-Paspuel and Garcia-Bereguiain, 2021b)
**Isopollo COVID-19 detection kit (Monitor, South Korea)**	NA	NA	NA	63.4%	100%	100,000 500	**Ecuador**	(Freire-Paspuel and Garcia-Bereguiain, 2021a)
**GenomeCoV19 kit** **(ABM, Canada)**	100%	100%	1 copy/µl of RNA extraction solution	75.0%	100%	8,000 40	**Ecuador**	(Freire-Paspuel and Garcia-Bereguiain, 2021c)
**Allplex 2019-nCoV Assay (Seegene, South Korea)**	100%	93.07%	12.5 RNA c/µl of RNA extraction solution	87.7%–98.2%	93.75%–100%	2,000–4,000 10–20	S.Korea/FDA	(Hur et al., 2020; Freire-Paspuel and Garcia-Bereguiain, 2021b; Hernandez et al., 2021; Liotti et al., 2021)
**Viasure SARS-CoV-2** **(CerTest Biotec, Spain)**	100%	97.5%	4 copies/µl of RNA extraction	91.86%	100%	2,000 10	Spain	(Freire-Paspuel et al., 2021c)
**COVID-19 (SARS-CoV-2) Nucleic Acid Test Kit (eDiagnosis, China)**	95.93%	94.07%	500 copies/ml of sample	100%	94.1%–100%	500 2.5	China	(Hernandez et al., 2021; Freire-Paspuel et al., 2021)
**Novel Coronavirus (2019-nCoV) Nucleic Acid Diagnostic Kit** **(Sansure Biotech, China)**	100%	100%	200 copies/ml of sample	83.3%–95.3%	87.5%–100%	484–3,000 0.66-5	China/FDA	(Iglói et al., 2020; Wang et al., 2020; Xiong et al., 2020; Yu et al., 2020; Freire-Paspuel et al., 2021)
**COVID-19 RT-PCR Real TM Fast (Cy5) (ATGen)**	NA	NA	NA	96.4%	96%–100%	2,000 10	Uruguay	(Freire-Paspuel et al., 2022)
**Detection Kit for 2019 Novel Coronavirus (2019-nCoV) RNA** **(Da An Gene, China)**	100%	100%	500 copies/ml of sample	78.6%–100%	100%	484–3,000 0.16–10	China	(Iglói et al., 2020; Wang et al., 2020; Xiong et al., 2020; Yu et al., 2020; Freire-Paspuel and Garcia-Bereguiain, 2021c)
**ECUGEN SARS-CoV-2 RT-PCR kit** **(UDLA-Starnewcorp; Ecuador)**	97.7%	100%	5 copies/µl of RNA extraction	100%	94.6%–100%	500–1,000 2.5–5	Ecuador	(Freire-Paspuel et al., 2022)

* Fellner et al., 2021 reports 100% sensitivity but is not a clinical performance evaluation as only artificial SARS-CoV-2 samples were used.

Sensitivity, specificity, and the limit of detection reported by manufacturers and by scientific publications are detailed (EUA, Emergency Use Authorization at the country of production and by the FDA; FDA, US Food and Drug Administration; Ref, references; NA, not available).

Unfortunately, the Ecuador’s government agency responsible for clinical use authorization lacks infrastructure to carry out clinical performance evaluations like the ones carried out in research laboratories. Moreover, local clinical microbiology laboratories without prior experience in PCR-based diagnosis began to perform SARS-CoV-2 detection by RT-qPCR, but, in general, those laboratories did not have the expertise to conduct internal evaluations of SARS-CoV-2 NAATs kits.

On the other hand, our research laboratory had previously reported several evaluations of new protocols related to the SARS-CoV-2 laboratory diagnosis, including studies addressing the use of cotton swabs for sample collection, an efficient high-throughput system with sample pooling prior to RT-qPCR, the clinical performance of an RNA-extraction free approach for SARS-CoV-2 diagnosis or a novel multiplex RT-qPCR method (Freire-Paspuel et al., 2020; Freire-Paspuel et al., 2020b; Bruno et al., 2021; Freire-Paspuel and Garcia-Bereguiain, 2021d; Ramirez-Cordova et al., 2023). The same approach was utilized for the clinical performance evaluation of SARS-COV-2 NAATs kits. The SARS-CoV-2 diagnosis gold-standard protocol used in our evaluations was nasopharyngeal swab sample collection followed by manual RNA extraction with column-based commercial kits prior to the detection of SARS-CoV-2 using the CDC-designed “FDA EUA 2019-nCoV CDC kit” (IDT, USA) (Freire-Paspuel et al., 2020; Freire-Paspuel et al., 2020b; Bruno et al., 2021; Freire-Paspuel and Garcia-Bereguiain, 2021d; Ramirez-Cordova et al., 2023). This protocol targets the conserved SARS-CoV-2 N1 and N2 sections of the viral N gene and uses the transcript for the human RNase P as an RNA extraction quality control; it is considered a gold standard for SARS-CoV-2 RT-qPCR diagnosis worldwide (Corman et al., 2020; Lu et al., 2020; PAHO, 2020). We additionally used “TaqMan 2019-nCoV Assay Kit v1” (Thermofisher, USA) as a reference method of a high-quality SARS-CoV-2 NAATs kit with EUA by the FDA.

Having said all the above, we herein present a comparative analysis of previously published results about the clinical performance and analytical sensitivity of 11 SARS-CoV-2 NAATs kits available in Ecuador. This comparative analysis is based on several peer-reviewed publications, including eight research articles from our laboratory. As detailed in **Table 1**, there were worrisome differences in the clinical performance among those 11 SARS-CoV-2 NAATs kits with sensitivity values ranging from 63.4% to 100%, compared to the gold standard, and the limit of detection ranging from 500 to 100,000 copies/ml. Noteworthy, five of those SARS-CoV-2 NAATs kits yielded substantially lower sensitivity (63.4%–78.9%) and a defective limit of detection (8,000–100,000 copies/ml) than that reported by manufacturers or to that recommended for a reliable SARS-CoV-2 diagnosis (Freire-Paspuel et al., 2020; Freire-Paspuel et al., 2020a; Freire-Paspuel and Garcia-Bereguiain, 2021a; Freire-Paspuel and Garcia-Bereguiain, 2021b; Freire-Paspuel et al., 2021). For the other seven NAATs kits, the available peer-reviewed publications reported a great clinical performance with sensitivity values ranging from 87.7% to 100% and the limit of detection of 500–4,000 copies/ml (Freire-Paspuel et al., 2022; Hur et al., 2020; Iglói et al., 2020; Shen et al., 2020; Wang et al., 2020; Xiong et al., 2020; Yu et al., 2020; Fellner et al., 2021; Freire-Paspuel and Garcia-Bereguiain, 2021b; Freire-Paspuel and Garcia-Bereguiain, 2021c; Freire-Paspuel et al., 2021; Freire-Paspuel et al., 2021; Hernandez et al., 2021; Liotti et al., 2021), in close concordance with the values reported by manufacturers. Two of the evaluation studies reported different values of sensitivity for two of the kits included in **Table 1** also evaluated in our laboratory (Salinas et al., 2022; Fellner et al., 2021). However, one of those studies is not properly a clinical evaluation because only artificial SARS-CoV-2 samples from viral cultures were used (Fellner et al., 2021). Moreover, the reference methods used in this two studies had a lower sensitivity that the gold standard method used in the evaluation carried out in our laboratory, resulting in a potential bias due to the exclusion of low-viral-load samples in those evaluations (Salinas et al., 2022; Fellner et al., 2021).

## Emergency use authorization at country of manufacture as a proxy for quality control for SARS-CoV-2 nucleic acid amplification tests in low- and middle-income countries

It was not surprising that the seven SARS-CoV-2 NAATs kits with great clinical performance had been granted with EUA in their country of origin, including EUA granted by the FDA in some cases, whereas those five kits with poor clinical performance lack of EUA at their country of manufacture (Freire-Paspuel and Garcia-Bereguiain, 2020; Freire-Paspuel and Garcia-Bereguiain, 2021a; Freire-Paspuel and Garcia-Bereguiain, 2021b; Freire-Paspuel et al., 2021).

Based on these findings, we propose to implement ethically correct public health policies in low- and middle-income countries aim to authorize the commercialization and use of reliable SARS-CoV-2 NAATs kits to those at least obtaining EUA in the country where the manufacturer has its headquarters. This would be particularly relevant for countries like Ecuador, where the local public health authorities did not carry out experimental evaluations to grant EUA to SARS-CoV-2 NAATs kits. While we acknowledge that these policies should be encouraged, designed, and implemented in-country, ignoring the fact that the issue did exist could have caused unnecessary morbidity and mortality in the affected countries and potentially negative side effects to the control of the COVID-19 pandemic in industrialized countries. For example, to travel to the USA from Ecuador, a negative SARS-CoV-2 NAATs result was requested during 2020–2022. If the SARS-CoV-2 NAATs kit utilized was, for instance, “Isopollo” (see **Table 1** for details), then nearly 4 out of 10 SARS-CoV-2 positive travelers would be allowed to enter the country, with a negative impact in the control of the COVID-19 pandemic.

## Cheap and sensitive SARS-CoV-2 NAATs developed at low- and middle-income countries as an alternative to the non-equitable distribution of good-quality SARS-CoV-2 NAATs

We call the attention of two of the SARS-CoV-2 NAATss kits detailed in **Table 1**. “COVID-19 RT-PCR Real TM FAST (CY5)” and “ECUGEN SARS-CoV-2 RT-qPCR” RT-PCR kits are within the group of SARS-CoV-2 NAATs kits with great clinical performance and analytical sensitivity (Freire-Paspuel et al., 2022). Moreover, both of them have equivalent clinical performance than TaqMan 2019-nCoV Assay Kit v1 (Thermo Fisher, USA), a reference of high-quality SARS-CoV-2 NAATs kits (Freire-Paspuel et al., 2022). Both kits are produced in South American countries (Uruguay and Ecuador) and were designed by a consortium between universities and private companies, pointing out the role of the academia to improve SARS-CoV-2 testing, as it has been suggested even in the USA (Mascuch et al., 2020). This is particularly relevant in the context of low- and middle-income countries, as high-quality locally produced SARS-CoV-2 NAATs kits would potentially increase SARS-CoV-2 testing capacities by two main reasons: (a) testing cost reduction as these local SARS-CoV-2 NAATs kits are substantially cheaper than “high-quality” imported ones and (b) SARS-CoV-2 testing supply shortages would be more easily overcome as local production is guaranteed. The local production of reagents and enzymes for SARS-CoV-2 NAATss has already been proposed by others and showed excellent results (Mascuch et al., 2020; Wozniak et al., 2020; Graham et al., 2021), and, although improving a good-quality control strategy for those local products would be mandatory, we believe that this should be the path to follow for a low-cost diagnosis in low- and middle-income countries.

## Conclusion: lessons for future pandemics

The COVID-19 pandemic was the worst public health threat that our globalized humanity has faced over the last few decades and needed a global health approach to be defeated. This is also true for future pandemics. This global health approach means that diagnostic quality and testing capacities should be guaranteed for any country in the world. Beyond a matter of human rights, it is the only way to fight infectious disease outbreaks and that should be one of the main lessons learned after the COVID-19 pandemic.

In summary, we suggest to Ecuadorian public health authorities to review the protocols for the EUA of SARS-CoV-2 NAATs kits that were adopted during the COVID-19 pandemic. We also express our concern to companies from high-income countries that were exporting low-quality products to low- and middle-income countries during the COVID-19 pandemic. We encourage the scientific community in low- and middle-income countries to carry out clinical performance evaluation studies for commercial diagnostic kits and publish their results, contributing as sentinels for quality control diagnosis in those settings. Finally, this letter is a call for action to international public health organizations to claim for a fair trade of SARS-CoV-2 NAATs kits and any diagnosis tool in general, based on universal quality standards without income bias.

## Author contributions

All the authors contributed to the conceptualization and writing of the manuscript. All the authors also contributed to the clinical performance evaluation studies previously published by our research team.
